# The association between multimorbidity and hospitalization is modified by individual demographics and physician continuity of care: a retrospective cohort study

**DOI:** 10.1186/s12913-016-1415-5

**Published:** 2016-04-27

**Authors:** Andrea Gruneir, Susan E. Bronskill, Colleen J. Maxwell, Yu Qing Bai, Anna J. Kone, Kednapa Thavorn, Yelena Petrosyan, Andrew Calzavara, Walter P. Wodchis

**Affiliations:** Department of Family Medicine, University of Alberta, 6-40 University Terrace, Edmonton, Alberta T6G 2T4 Canada; Institute for Clinical Evaluative Sciences, 2075 Bayview Avenue, G-Wing, Toronto, Ontario M4N 3M5 Canada; Institute of Health Policy Management & Evaluation, University of Toronto, 155 College Street, 4th Floor, Toronto, Ontario M5T 3M6 Canada; School of Pharmacy, University of Waterloo, 200 University Avenue West, Waterloo, Ontario N2L 3G1 Canada; Cancer Care Ontario, 620 University Ave, Toronto, Ontario M5G 2L7 Canada; Ottawa Hospital Research Institute, The Ottawa Hospital, 501 Smyth Road, PO Box 201B, Ottawa, Ontario K1H 8L6 Canada

**Keywords:** Chronic conditions, Age, Sex and gender, Continuity of care, Readmission

## Abstract

**Background:**

Multimorbidity poses a significant clinical challenge and has been linked to greater health services use, including hospitalization; however, we have little knowledge about the influence of contextual factors on outcomes in this population. Objectives: To describe the extent to which the association between multimorbidity and hospitalization is modified by age, gender, primary care practice model, or continuity of care (COC) among adults with at least one chronic condition.

**Methods:**

A retrospective cohort study with linked population-based administrative data.

Setting: Ontario, Canada. Cohort: All individuals 18 and older with at least one of 16 priority chronic conditions as of April 1, 2009 (baseline). Main Outcome Measures: Any hospitalization, 3 or more hospitalizations, non-medical discharge delay, and 30-day readmission within the 1 year following baseline.

**Results:**

Of 5,958,514 individuals, 484,872 (8.1 %) experienced 646,347 hospitalizations. There was a monotonic increase in the likelihood of hospitalization and related outcomes with increasing multimorbidity which was modified by age, gender, and COC but not primary care practice model. The effect of increasing multimorbidity was greater in younger adults than older adults and in those with lower COC than with higher COC. The effect of increasing multimorbidity on hospitalization was greater in men than women but reversed for the other outcomes.

**Conclusions:**

The effect of multimorbidity on hospitalization is influenced by age and gender, important considerations in the development of person-centred care models. Greater continuity of physician care lessened the effect of multimorbidity on hospitalization, further demonstrating the need for care continuity across providers for people with chronic conditions.

**Electronic supplementary material:**

The online version of this article (doi:10.1186/s12913-016-1415-5) contains supplementary material, which is available to authorized users.

## Background

Multimorbidity, the co-occurrence of two or more chronic conditions, is an important issue in clinical care and population health [1, 2]. Several studies have demonstrated strong associations between the burden of multimorbidity and health services use, especially hospitalizations [3–6]. Addressing the needs of individuals with multimorbidity continues to be a vexing clinical challenge due in part to the lack of appropriate evidence-based guidelines [1]. Patient care is also challenged by health systems that are organized around health care providers focused on single conditions with payment systems that reinforce that model [7]. This mismatch between population needs and health system design has prompted calls for person-centred care practices with a renewed focus on primary care [8–10].

Important gaps persist in our understanding of how patient and health system context influence service utilization and outcomes for people with multimorbidity. Context encompasses the range of biological, social, environmental, and health system factors that impact on health but that are often ignored or “adjusted” in research on multimorbidity [11]. A prime example is the treatment of age and gender – two fundamental individual characteristics that are associated with multimorbidity and health care use [5, 12] but have received little attention beyond treatment as confounding variables.

There is also little evidence regarding the role of health system factors, a significant omission given the potential for alternate health care provider arrangements, including physician payments and access to interdisciplinary resources, to facilitate patient-centred care. Moreover, specialists play an important role in the care of individuals with chronic conditions, and their use increases with the number of chronic conditions [13] This has implications for continuity of care, which has been shown to improve outcomes; although the same has yet to be investigated in the context of multimorbidity where involvement of a greater number of specialists can be expected.

The purpose of this study is to explore the role of key individual demographic and health system contextual factors in influencing the association between multimorbidity and hospitalization. Our objectives are to describe hospitalizations and related outcomes among a population-based cohort of adults by their degree of multimorbidity, and to test whether age, gender, primary care practice model, or continuity of care modify the association between multimorbidity and hospitalization. This research should help to target strategies to high-risk groups and suggest system responses to mitigate the impact of multimorbidity. We also aim to demonstrate the importance of incorporating context into the study of multimorbidity, an issue that has been highlighted by others as critical to moving the field forward [11].

## Methods

### Setting

This study was conducted in Ontario, Canada’s largest province with approximately 13 million residents. The provincial health insurance program insures virtually all residents for physician and hospital care.

### Study design and data

This is a retrospective cohort study conducted using linked population-based administrative data. These data include: the Registered Persons Database for demographic information on all residents with provincial health insurance; the Discharge Abstract Database (DAD) for data from all hospital discharges; the Ontario Health Insurance Plan (OHIP) claims database for physician billing claims; and the Client Agency Program Enrolment database for patient enrolment to primary care physicians and the practice model type. These data are regularly used in research [14–16].

The data were linked using unique encoded identifiers and are maintained at the Institute for Clinical Evaluative Sciences (ICES), where the data were analyzed. This study was approved by the Research Ethics Board at Sunnybrook Health Sciences Centre.

We included all Ontario residents over the age of 18 years with one of 16 chronic conditions as of April 1, 2009 (baseline): acute myocardial infarction, asthma, cancer, cardiac arrhythmia, chronic obstructive pulmonary disease, congestive heart failure, coronary syndrome, dementia, diabetes, hypertension, mood disorders, osteoarthritis, osteoporosis, renal failure, rheumatoid arthritis, or stroke. We selected these conditions based on prevalence and system burden [17–20]. Each condition was defined as the presence of two physician billing codes within 2 years or one hospital diagnostic code anytime between April 1, 2001 and baseline (or between April 1, 2007 and baseline for cancer or mood disorders) (See Appendix). We categorized the cohort as having one through five or more conditions at baseline. We used a simple count since there are no existing methods for creating meaningful clusters, especially without a central index condition. We included those with a single condition, even though this is not considered multimorbidity, to characterize the increasing risk of hospitalization with additional chronic conditions.

### Hospitalizations

We followed each cohort member from baseline for up to 1 year and captured all unplanned hospital episodes. We examined four measures of hospital use within the year: 1) any hospitalization; 2) three or more hospitalizations; 3) hospitalizations with non-medical discharge delay; and 4) 30-day hospital readmission following discharge. For each hospital episode, we characterized the most responsible diagnosis for the admission, the length of stay, and discharge due to death. We also determined if the most responsible diagnosis for the admission matched a pre-existing condition recorded at baseline, an incident condition, or another reason. Non-medical discharge delays were identified using “alternate level of care” days which refer to periods when a patient no longer requires hospital services but cannot be discharged due to inadequate services elsewhere (for example, when a long-term care bed is required but not available) [21]. Hospital readmissions were counted only among those discharged alive.

### Contextual factors

The contextual factors were: age, gender, primary care practice model, and continuity of care. The cohort was stratified as 18–64 years and 65–105 years. We chose this simple age stratification since this is a preliminary exploration on the differences between younger and older adults.

The broad distinguishing characteristics of the primary care practice models in Ontario are the degree of cooperation across physicians, the integration of other providers, and reimbursement type, which lead to different incentives and care practices [22]. Based on funding and interdisciplinary access, we created a three-level variable to describe the primary care practice model: a) non-capitated includes all non-rostered models where physicians largely operate on a fee-for-service basis; b) capitated includes models funded primarily via an age-sex adjusted capitation scheme; and c) capitated + includes models which operate similar funding to the capitated group but with additional payments for interdisciplinary care [23]. We anticipated that the association between multimorbidity and hospitalization would be weaker among individuals who belong to capitated and capitated + primary care practice models because of the greater emphasis on chronic condition management.

Continuity of care was measured using the Bice Concentration of Care Index (COC), which is an expression of the dispersion of physician visits over time across the number of individual physicians visited [24]. The index has a maximum score of 1.0 if all visits are to one physician and approaches zero with additional physicians visited; it accounts for the increase in the number of visits associated with an increasing number of physicians involved in care. We calculated the COC using billing codes for any outpatient physician visit, including all primary and specialty care visits, over the year prior to baseline. We included both primary care and specialist visits since individuals with chronic conditions may receive a significant share of their care from specialists [25] and since specialists are often involved in the management of common conditions [13]. Using the cohort median COC score (0.52), we categorized the cohort as either having a high concentration of care (greater continuity) or a low concentration (less continuity) and anticipated that higher COC would be protective against hospitalization and related outcomes.

### Analyses

We described the frequency of hospitalization by number of chronic conditions. We used logistic regression to estimate the association between the degree of multimorbidity and each of: any hospitalization, three or more hospitalizations, non-medical discharge delay, and hospital readmission within 30 days of discharge during the follow-up. The likelihood of any hospitalization was modeled for the entire cohort but the likelihood of three or more hospitalizations and non-medical discharge delay was modeled only among those with at least one hospitalization. The likelihood of 30-day readmission was modeled only among those discharged alive from a prior hospitalization.

To assess for effect measure modification, we created a set of mutually exclusive variables to cross-classify individuals by degree of multimorbidity and each contextual factor. For example, we classified each individual as: age < 65 years with one condition, age < 65 years with two conditions, etc. We created a total of 16 logistic regression models (one per contextual factor per outcome). This strategy allows for direct estimation of the effect of multimorbidity on outcomes at each level of the contextual factor [26] so that we could directly compare odds ratios within and between levels of the contextual factor. The reference categories were set as those anticipated to have the lowest risk for hospitalization: younger age (18–64 years), female, capitated + primary care practice model, and higher COC. The models testing age and gender were unadjusted for other variables but the models testing primary care practice model and continuity of care were adjusted for age and gender.

All analyses were completed using SAS version 9.3.

## Results

We identified 5,958,514 individuals with at least one of 16 conditions. Nearly 52 % of individuals had two or more conditions and 5.8 % had five or more (Table 1). Mean age for the cohort was 53.0 (standard deviation = 18.0) years but this varied from 44.9 (SD = 15.8) years among those with one condition to 73.6 (SD = 11.9) among those with five or more. Fifty-five percent of the cohort was female. The most prevalent priority conditions were osteoarthritis (44.4 %), hypertension (43.4 %), and mood disorders (22.7 %). The majority were enrolled in a non-capitated primary care practice model (66.7 %). Continuity of care was relatively stable by degree of multimorbidity.

**Table 1:** Baseline characteristics of adults, aged 18 years and older, with at least one priority chronic condition, by burden of multimorbidity (Ontario, Canada, April 1, 2009)

	Number of priority chronic conditions at baseline					
	1	2	3	4	5+	Total
Number of Individuals^a^	2844033 (47.7)	1548197 (26.0)	812429 (13.6)	405766 (6.8)	348089 (5.8)	5958514 (100)
Age (in years), mean (SD)	44.9 (15.8)	54.5 (16.2)	62.4 (14.9)	69.0 (13.5)	73.6 (11.9)	53.0 (18.0)
Age Groups, N (%)						
<65 years	2523875 (88.7)	1124098 (72.6)	440940 (54.3)	157162 (38.7)	78049 (22.4)	4324124 (72.6)
≥65 years	320158 (11.3)	424099 (27.4)	371489 (45.7)	248604 (61.3)	270040 (77.6)	1634390 (27.4)
Female, N (%)	1477561 (52.0)	872671 (56.4)	472030 (58.1)	234217 (57.7)	194732 (55.9)	3251211 (54.6)
Prevalence of Each Priority Condition, N (%)						
Osteoarthritis	785297 (27.6)	777387 (50.2)	523568 (64.4)	289806 (71.4)	269393 (77.4)	2645451 (44.4)
Rheumatoid arthritis	11447 (0.4)	30963 (2.0)	34028 (4.2)	25879 (6.4)	32116 (9.3)	134433 (2.3)
Cancer	197688 (7.0)	226464 (14.6)	181622 (22.4)	116682 (28.8)	122160 (35.1)	844616 (14.2)
Arrhythmia	26284 (0.9)	44264 (2.7)	57281 (7.1)	59136 (14.6)	116922 (33.6)	303887 (5.1)
Dementia	8695 (0.3)	20556 (1.3)	29767 (3.7)	30878 (7.6)	58159 (16.7)	148055 (2.5)
Mood disorders	470962 (16.6)	401028 (25.9)	233008 (28.7)	125289 (30.9)	119660 (34.4)	1349947 (22.7)
Osteoporosis	41424 (1.5)	72486 (4.7)	70425 (8.7)	48836 (12.0)	52837 (15.2)	286008 (4.8)
Renal disease	5581 (0.2)	16568 (1.1)	27301 (3.4)	29958 (7.4)	68164 (19.6)	147572 (2.5)
Stroke	5549 (0.2)	15923 (1.0)	25059 (3.1)	26025 (6.4)	50319 (14.5)	122875 (2.1)
Coronary artery disease	33552 (1.2)	99472 (6.4)	147047 (18.1)	139099 (34.3)	205730 (59.1)	624900 (10.5)
Asthma	515746 (18.1)	323609 (20.9)	190172 (23.4)	112921 (27.8)	130775 (37.6)	1273223 (21.4)
Congestive heart failure	2309 (0.1)	10806 (0.7)	27568 (3.4)	44045 (10.9)	131444 (37.8)	216172 (3.6)
Chronic obstructive pulmonary disease	13215 (0.5)	33266 (2.2)	49300 (6.1)	52272 (12.9)	108959 (31.3)	257012 (4.3)
Hypertension	573702 (20.2)	756405 (48.7)	585695 (72.1)	346365 (85.4)	326183 (93.7)	2588350 (43.4)
Diabetes mellitus	152497 (5.4)	266126 (17.2)	253328 (31.2)	173209 (42.7)	190688 (54.8)	1035848 (17.4)
Acute myocardial infarction	85 (0.0)	1071 (0.1)	2118 (0.3)	2664 (0.7)	7093 (2.0)	13031 (0.2)
Primary Care Practice Model,^b^ N (%)						
Non-capitated	1921317 (67.6)	1020886 (65.9)	535298 (65.9)	267574 (65.9)	231673 (66.6)	3976748 (66.7)
Capitated	442850 (15.6)	258985 (16.7)	137301 (16.9)	68569 (16.9)	58198 (16.2)	965903 (16.2)
Capitated+	479866 (16.9)	268326 (17.3)	139830 (17.2)	69623 (17.2)	58218 (16.7)	1015863 (17.1)
Continuity of Care^c^						
Mean (SD)	0.6 (0.3)	0.7 (0.3)	0.7 (0.3)	0.7 (0.3)	0.6 (0.3)	0.6 (0.3)
Median (Q1-Q3)	0.5 (0.3–0.8)	0.5 (0.3–0.8)	0.5 (0.3–0.8)	0.5 (0.3–0.8)	0.5 (0.3–0.7)	0.5 (0.3–0.8)
High (>0.52), N (%)	1244438 (43.8)	802231 (51.8)	430849 (53.0)	203662 (50.2)	147651 (42.4)	2828831 (47.5)
Low (<=0.52), N (%)	1599595 (56.2)	745966 (48.2)	381580 (47.0)	202104 (49.8)	200438 (57.6)	3129683 (52.5)

^a^Parentheses show row percent

^b^Primary care practice models defined as: non-capitated models include non-rostered models and those that operate on a fee-for-service basis; capitated models include family health networks and family health organizations operating on a age-sex adjusted capitation funding scheme; and the capitated + models include family health teams and other rostered models operating on a capitated funding scheme with additional incentives for interdisciplinary care

^c^Calculated using the Bice Concentration of Care Index

In the year following baseline, 484,872 individuals (8.1 %) were hospitalized 646,347 times. The frequency of hospitalization and related outcomes increased markedly with multimorbidity (Table 2). The proportion with at least one hospitalization increased from 4.6 % among those with one condition to 26.9 % among those with five or more conditions. Among those hospitalized, 4.3 % with one condition experienced three or more hospitalizations compared to 13.8 % with five or more conditions. Hospitalizations with non-medical discharge delay, inpatient death, and 30-day readmission also increased with multimorbidity. The direct, unadjusted effect of multimorbidity on outcomes is shown in Table 3. In all cases, there is a clear monotonic increase in the ORs with tight 95 % CIs.

**Table 2:** Characteristics of all hospitalizations within 1 year for adults, aged 18 years and older with at least one priority chronic condition, Ontario, Canada, April 1, 2009 – March 31, 2010

	Number of priority chronic conditions					
	1	2	3	4	5+	Total
Number of Individuals	2844033	1548197	812429	405766	348089	5958514
Any hospitalization, N (%)	131029 (4.6)	108939 (7.0)	87273 (10.7)	64120 (15.8)	93511 (26.9)	484872 (8.1)
3+ hospitalizations, N (%^a^)	5581 (4.3)	6680 (6.1)	6893 (7.9)	6048 (9.4)	12907 (13.8)	38109 (7.9)
Hospitalizations						
Total Number of Hospitalizations	156507	138190	116481	89293	145876	646347
Hospitalization for an existing chronic condition, N (%^b^)	10,963 (7.0)	20,304 (14.7)	22,392 (19.2)	19,305 (21.6)	35,224 (24.1)	108,188 (16.7)
Hospitalization for a new chronic condition, N (%^b^)	14503 (9.3)	14700 (10.6)	12381 (10.6)	8949 (10.0)	14401 (9.9)	64934 (10.0)
Length of stay, days Median (IQR)	3 (1–5)	3 (2–6)	4 (2–8)	5 (2–9)	6 (3–11)	4 (2–7)
Non-medical discharge delay, N (%^b^)	4832 (3.1)	7769 (5.6)	9477 (8.1)	8901 (10.0)	17622 (12.1)	48601 (7.5)
Discharge due to death, N (%^b^)	3942 (3.0)	6138 (5.6)	7041 (8.1)	6667 (10.4)	14111 (15.1)	37899 (7.8)
30-day readmission among those discharged alive, N (%^c^)	9284 (7.3)	10076 (9.8)	9503 (11.8)	8171 (14.2)	15842 (20.0)	52876 (11.8)

*IQR* interquartile range

^a^% of individuals with any hospitalization; ^b^% of all hospitalizations; ^c^% of all hospitalizations where patient was discharged alive

**Table 3:** Direct effect of multimorbidity on the odds of hospitalization and related outcomes within 1 year among adults, aged 18 years and older with at least one priority chronic condition, by burden of multimorbidity, April 1, 2009 – March 31, 2010

	Number of chronic conditions				
	1	2	3	4	5+
Any hospitalization, OR (95 % CI)	Ref	1.6 (1.5–1.6)	2.5 (2.5–2.5)	3.9 (3.8–3.9)	7.6 (7.5–7.6)
Three or more hospitalizations among those hospitalized, OR (95 % CI)	Ref	1.6 (1.5–1.6)	2.1 (2.1–2.2)	2.7 (2.7–2.8)	4.3 (4.2–4.4)
Any days with non-medical discharge delay among those hospitalized, OR (95 % CI)	Ref	2.0 (2.0–2.1)	3.2 (3.1–3.3)	4.2 (4.1–4.4)	6.0 (5.9–6.2)
30-day readmission among those discharged alive, OR (95 % CI)	Ref	1.4 (1.4–1.4)	1.7 (1.7–1.8)	2.1 (2.0–2.2)	3.1 (3.1–3.2)

**Fig. 1:**
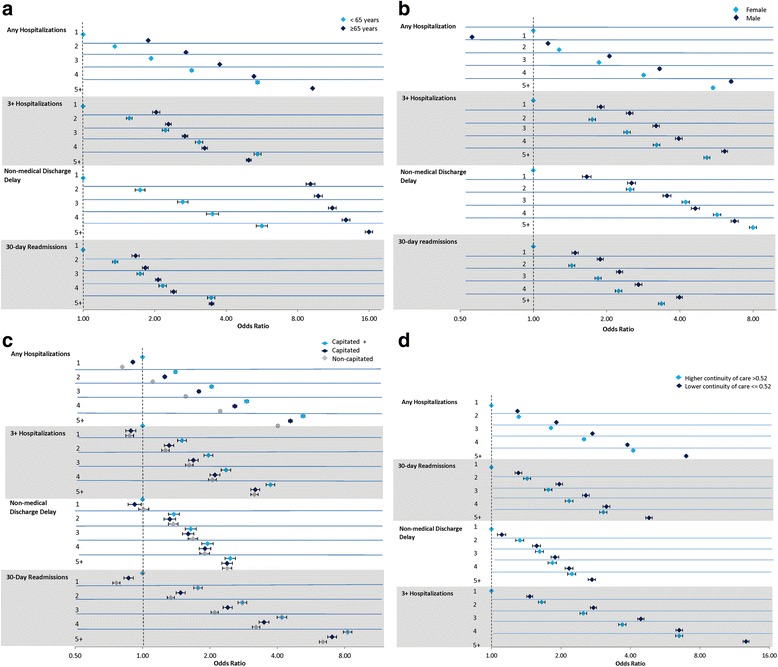
Association of multimorbidity and hospitalization and related outcomes within one year as modified by: **a**) age, **b**) gender, **c**) primary care model and d) continuity of care age, by burden of multimorbidity, April 1, 2009 - March 31, 2010

Figure 1a–d illustrates the ORs and 95 % CIs generated from cross-classifying multimorbidity with each contextual factor. To assess for effect measure modification, we compared the odds ratio of the five or more condition group against the odds ratio for the single condition group within each level of the contextual factor (for example, within younger adults) and then compared that ratio across levels of the contextual factor (between younger and older adults). Approximately 5.6 % of those under 65 were hospitalized at least once relative to 14.8 % of those over 65 years (not shown). Age appeared to modify the association between multimorbidity and hospitalization (Fig. 1a). In younger adults, the odds of hospitalization increased 5.4-fold from one condition to five or more conditions, whereas for older adults, the odds increased 4.8-fold with multimorbidity (younger adults: 5.4/1.0 vs. older adults: 9.2/1.9). Similar patterns emerged for the other outcomes. See Additional file 1 TableS1–S4 for the number and proportion of individuals who experienced each outcome by age, sex, primary care practice model and continuity of care.

Among those with a single condition, men had 40 % lower odds of hospitalization than women; however, the association reversed with multimorbidity (Fig. 1b). Among women, the odds of hospitalization increased five-fold with degree of multimorbidity; among men the same odds increased nearly 11-fold with degree of multimorbidity (women: 5.5/1.0 vs men: 6.5/0.6). Men showed greater odds of each three or more hospitalizations, non-medical discharge delay, and 30-day readmission among those with a single condition, but the relative increase in the odds with multimorbidity was consistently greater for women on all outcomes (even though the odds of three or more hospitalizations and 30-day readmission were higher for men at each level of multimorbidity).

After adjusting for age and gender, individuals in capitated and non-capitated primary care practice models showed lower odds of hospitalization, three or more hospitalizations, and 30-day readmissions compared to individuals in capitated + practice models. There was no difference in the relative odds of hospitalization or related outcomes with increasing multimorbidity across primary care practice models indicating no effect measure modification (Fig. 1c).

The modifying effect of COC was inconsistent across outcomes. Among those with low continuity (COC ≤ 0.52), the odds of hospitalization increased over five-fold with greater multimorbidity but only four-fold in those with high continuity (low continuity: 7.0/1.3 vs. high continuity: 4.1/1.0). A similar pattern emerged for 30-day readmissions but no such differences were observed for the other outcomes (Fig. 1d).

## Discussion

In this population-based study of nearly six million adults with at least one of 16 chronic conditions, we found a high prevalence of multimorbidity that was strongly associated with hospitalization. Our findings suggest that hospitalizations also become more complicated with increasing multimorbidity as demonstrated by longer stays, increased frequency of non-medical discharge delays and in-hospital death, as well as, a greater likelihood of multiple hospitalizations, including 30-day readmissions. These findings reinforce calls for better integration of patient-centred care practices both within and across health care sectors and the sharing of information to support care for those with multimorbidity.

We found that the association between multimorbidity and hospitalization was modified by both age and gender, two important contextual factors that are often overlooked. The prevalence of multimorbidity increases with age and much of the literature focuses on older adults, highlighting its importance in older populations, [5] but in our study, as in others, there were more younger adults than older adults living with multimorbidity [27, 28]. Further, the impact of increasing multimorbidity was relatively greater in younger adults than in older adults even though older adults were consistently at greater risk of hospitalization and related outcomes. The reason for the differential effect is unknown but may result from different clustering of chronic conditions by age and/or a degree of vulnerability inherent to having multiple chronic conditions at a young age that results in worse relative symptoms and outcomes.

The impact of gender on the association between multimorbidity and hospitalization was not consistent across outcomes. Men were more likely to be affected by multimorbidity when it came to hospitalization but women were more likely to be affected by multimorbidity on all other outcomes. This is likely attributed to underlying social differences, such as income, living arrangements, and access to informal caregivers, which tend to put women, especially older women, at greater risk for requiring supportive care services [29]. While some research shows that women experience a higher burden of multimorbidity, this has not been consistent [12, 30] and it is not yet clear how this relates to health services utilization given that men and women also have different patterns of health seeking behaviour.

A recent publication details the contextual factors identified by stakeholders as critical to moving the research agenda on multimorbidity forward [11]. Neither age nor gender were explicitly identified among that long list even though both cut across their broad categories of biological, person, and family factors. Our results demonstrate the importance of considering the moderating impact of age and gender and the influence of different biological, health behaviour, and social structures on health outcomes among people with multimorbidity. Future research should also consider the intersection of these factors as well as others, such as ethnicity and socioeconomic standing [31].

With respect to health system factors, the results were mixed. Attachment to a capitated + primary care practice model was associated with poorer outcomes than other models and there was no evidence of effect modification. It is difficult to know why this pattern emerged. Some research suggests that practice models such as those in the capitation + group have a higher case-mix of chronic conditions and multimorbidity than other practices, [32] meaning that preventing hospitalization may be more challenging among their patients. One Ontario-based evaluation found more emergency department visits among patients in capitated models but suggested that this was due to patterns of use that pre-dated funding changes [22]. Alternatively, and perhaps most importantly, primary care practice model may be a poor proxy for quality of care and future research should focus on specific aspects of service delivery.

Continuity of care, measured across all the physicians visited over 1 year, did modify the association between multimorbidity and hospitalization. The effect of multimorbidity on hospitalization, multiple hospitalizations, and 30-day readmissions was less pronounced among individuals with greater continuity than those with lesser continuity. The same was not observed for non-medical discharge delays, likely because of different health system drivers. The Bice Index is a proxy for relationship continuity based solely on the dispersion of visits across physicians and does not account for other aspects such as informational continuity [33]. Regardless, we demonstrate that when visits are concentrated with a single physician, even among individuals with multimorbidity, the risk of hospitalization is reduced. The magnitude was particularly remarkable as individuals with less than median COC experienced outcomes that were equivalent to those with high continuity with one less chronic condition. The benefits of having one provider who can provide oversight for patients with complex needs has been demonstrated by others [34, 35] and has been reflected in upcoming changes to the U.S. Medicare fee schedule [7].

This study has limitations. First, we focused on 16 conditions, likely underestimating the burden of multimorbidity; however, these are highly prevalent conditions and are consistent with those used to report on multimorbidity elsewhere [6]. Second, there is the potential for diagnostic misclassification due to the administrative data. Although many of our diagnostic algorithms have high sensitivity and specificity, others do not. Third, we used a simple approach to operationalizing multimorbidity by strictly counting conditions and could not account for severity or symptom burden. Our diagnostic count does not address concordant, discordant, or other combinations used to examine co-morbidity in specific diagnostic groups [36]. However, with no obvious method for clustering conditions, a simple diagnostic counts is the most straightforward method to define multimorbidity across population-based studies such as ours.

## Conclusions

We found that demographic and modifiable health system factors influenced the impact of multimorbidity on the risk of hospitalization and related outcomes. Our findings on age and gender help to identify groups that may be at particularly high risk for adverse outcomes associated with multimorbidity; however, additional research is required to better characterize this vulnerability including attention to other demographic factors and the mechanisms through which they influence outcomes. Our findings also support the importance of continuity of care for complex patients. Chronic condition management programs must incorporate levers to promote continuity, especially for patients who are likely to see multiple providers. While primary care is the obvious setting for coordinated and integrated care, our study was unable to determine differences across practice model types. Additional research on key aspects of chronic condition management in primary care and the impact on people with multimorbidity are still required.

## Availability of data and materials

Data are available from the Institute for Clinical Evaluative Sciences for researchers who meet the criteria for access to confidential data.

## Appendix

**Table 4:** List of diagnosis codes for defining the 16 selected conditions

Condition	ICD 9/OHIP	ICD 10
AMI	410	I21, I22
Arthritis - Osteoarthritis	715	M15-M19
Arthritis - Other Arthritis (Synovitis, Fibrositis, Connective tissue disorders, Ankylosing spondylitis, Gout Traumatic arthritis, pyogenic arthritis, Joint derangement, Dupuytren’s contracture, Other MSK disorders)	727, 729, 710, 720, 274, 716, 711, 718, 728, 739	M00-M03, M07, M10, M11-M14, M20-M25, M30-M36, M65-M79
Arthritis - Rheumatoid arthritis	714	M05-M06
Asthma	493	J45
Cancer	140-239	C00-C26, C30-C44, C45-C97,
Cardiac Arrythmia	427.3 (DAD)/427 (OHIP)	I48.0, I48.1
CHF	428	I500, I501, I509
COPD	491, 492, 496	J41, J43, J44
Dementia	290, 331, 797 (OHIP)/290.0, 290.1, 290.3, 290.4, 290.8, 290.9, 294.1, 294.8, 294.9, 331.0, 331.1, 331.2, 797 (DAD)	F000, F001, F002, F009, F010, F011, F012, F013, F018, F019, F020, F021, F022, F023, F024, F028, F03, F051, F065, F066, F068, F069, F09, G300, G301, G308, G309, G310, G311, R54
Depression	311, 300, 296	F32, F33, F412, F480
Diabetes	250	E08 - E13
Hypertension	401, 402, 403, 404, 405	I10, I11, I12, I13, I15
Osteoporosis	733	M81 M82
Renal failure	403,404,584,585,586,v451	N17, N18, N19, T82.4, Z49.2, Z99.2
Stroke	430, 431, 432, 434, 436	I60-I64
Coronary syndrome (excluding MI)	411-414	I20, I22-I25

## Additional file

Additional file 1: Supplementary Tables. **Table S1.** Number and proportion of individuals who experienced each outcome, stratified by age and number of priority chronic conditions (column percent shown). **Table S2.** Number and proportion of individuals who experienced each outcome, stratified by gender and number of priority chronic conditions (column percent shown). **Table S3.** Number and proportion of individuals who experienced each outcome, stratified by primary care practice model and number of priority chronic conditions (column percent shown). **Table S4.** Number and proportion of individuals who experienced each outcome, stratified by continuity of care and number of priority chronic conditions (column percent shown). (DOCX 147 kb)

## Abbreviations

COC: continuity of care
ICES: Institute for Clinical Evaluative Sciences
IQR: interquartile range
