# Supplementary material for: The association between multimorbidity and hospitalization is modified by individual demographics and physician continuity of care: a retrospective cohort study
Source: BMC Health Serv Res. 2016 Apr 27;16:154. doi: 10.1186/s12913-016-1415-5 (PMC4848783; doi:10.1186/s12913-016-1415-5)
Supplement: Additional file 1: — Supplementary Tables. Table S1. Number and proportion of individuals who experienced each outcome, stratified by age and number of priority chronic conditions (column percent shown). Table S2. Number and proportion of individuals who experienced each outcome, stratified by gender and number of priority chronic conditions (column percent shown). Table S3. Number and proportion of individuals who experienced each outcome, stratified by primary care practice model and number of priority chronic conditions (column percent shown). Table S4. Number and proportion of individuals who experienced each outcome, stratified by continuity of care and number of priority chronic conditions (column percent shown). (DOCX 147 kb) [file 12913_2016_1415_MOESM1_ESM.docx]

Supplementary Table 1: Number and proportion of individuals who experienced each outcome, stratified by age and number of priority chronic conditions (column percent shown)

|  | <65 years | | | | | ≥65 years | | | | |
| --- | --- | --- | --- | --- | --- | --- | --- | --- | --- | --- |
|  | Number of Priority Chronic Conditions | | | | | Number of Priority Chronic Conditions | | | | |
|  | 1 | 2 | 3 | 4 | 5+ | 1 | 2 | 3 | 4 | 5+ |
| Number of Individuals | 2523875 | 1124098 | 440940 | 157162 | 78049 | 320158 | 424099 | 371489 | 248604 | 270040 |
| Any hospitalization, N (%) | 108060 (4.28) | 65495 (5.83) | 36106 (8.19) | 18525 (11.79) | 16159 (20.7) | 23357 (7.3) | 43865 (10.34) | 51534 (13.87) | 45842 (18.44) | 77643 (28.75) |
| 3+ hospitalizations, N (%*) | 4128 (3.82) | 3798 (5.8) | 2913 (8.07) | 1916 (10.34) | 2563 (15.86) | 1676 (7.18) | 3265 (7.44) | 4418 (8.57) | 4558 (9.94) | 11064 (14.25) |
| Non-medical discharge delay, N (%*) | 1653 (1.53) | 1698 (2.59) | 1433 (3.97) | 966 (5.21) | 1221 (7.56) | 2926 (12.53) | 5732 (13.07) | 7477 (14.51) | 7349 (16.03) | 14882 (19.17) |
| 30-day readmission, N (%**) | 6923 (0.27) | 5447 (0.48) | 3666 (0.83) | 2222 (1.42) | 2721 (3.49) | 2077 (0.65) | 4094 (0.97) | 5160 (1.39) | 5278 (2.13) | 11410 (4.25) |

*Among those hospitalized; **Among those discharged alive

Supplementary Table 2: Number and proportion of individuals who experienced each outcome, stratified by gender and number of priority chronic conditions (column percent shown)

|  | Women | | | | | Men | | | | |
| --- | --- | --- | --- | --- | --- | --- | --- | --- | --- | --- |
|  | Number of Priority Chronic Conditions | | | | | Number of Priority Chronic Conditions | | | | |
|  | 1 | 2 | 3 | 4 | 5+ | 1 | 2 | 3 | 4 | 5+ |
| Number of Individuals | 1477561 | 872671 | 472030 | 234217 | 194732 | 1366472 | 675526 | 340399 | 171549 | 153357 |
| Any hospitalization, N (%) | 85760 (5.8) | 64208 (7.36) | 48950 (10.37) | 35069 (14.97) | 49754 (25.55) | 45657 (3.34) | 45152 (6.68) | 38690 (11.37) | 29298 (17.08) | 44048 (28.72) |
| 3+ hospitalizations, N (%*) | 3251 (3.51) | 3671 (5.59) | 3694 (7.51) | 3212 (9.15) | 6658 (13.38) | 3376 (6.27) | 3710 (7.97) | 3725 (9.57) | 3287 (11.2) | 6975 (15.83) |
| Non-medical discharge delay, N (%*) | 2448 (2.85) | 4377 (6.82) | 5332 (10.89) | 4946 (14.1) | 9158 (18.41) | 2131 (4.67) | 3053 (6.76) | 3578 (9.25) | 3369 (11.5) | 6945 (15.77) |
| 30-day readmission, N (%**) | 5202 (0.35) | 5149 (0.59) | 4575 (0.97) | 3859 (1.65) | 7083 (3.66) | 3798 (0.28) | 4392 (0.65) | 4251 (1.25) | 3641 (2.13) | 7048 (4.63) |

*Among those hospitalized; **Among those discharged alive

Supplementary Table 3: Number and proportion of individuals who experienced each outcome, stratified by primary care practice model and number of priority chronic conditions (column percent shown)

|  | Capitated+ | | | | | Capitated | | | | | Non-Capitated | | | | |
| --- | --- | --- | --- | --- | --- | --- | --- | --- | --- | --- | --- | --- | --- | --- | --- |
|  | Number of Priority Chronic Conditions | | | | | Number of Priority Chronic Conditions | | | | | Number of Priority Chronic Conditions | | | | |
|  | 1 | 2 | 3 | 4 | 5+ | 1 | 2 | 3 | 4 | 5+ | 1 | 2 | 3 | 4 | 5+ |
| Number of Individuals | 479866 | 268326 | 139830 | 69623 | 58218 | 442850 | 258985 | 137301 | 68569 | 58198 | 1921317 | 1020886 | 535298 | 267574 | 231673 |
| Any hospitalization, N (%) | 26237 (5.47) | 22835 (8.51) | 18138 (12.97) | 13136 (18.87) | 17861 (30.68) | 21631 (4.88) | 19687 (7.6) | 15916 (11.59) | 11737 (17.12) | 16289 (27.99) | 83549 (4.35) | 66838 (6.55) | 53586 (10.01) | 39494 (14.76) | 59652 (25.75) |
| 3+ hospitalizations, N (%*) | 1444 (5.04) | 1689 (7.25) | 1748 (9.61) | 1446 (11) | 2842 (15.91) | 1027 (4.35) | 1320 (6.57) | 1372 (8.59) | 1159 (9.87) | 2338 (14.35) | 4156 (4.41) | 4372 (6.35) | 4299 (7.97) | 3894 (9.85) | 8453 (14.17) |
| Non-medical discharge delay, N (%*) | 1030 (3.93) | 1722 (7.54) | 1899 (10.47) | 1760 (13.4) | 3129 (17.52) | 710 (3.28) | 1426 (7.24) | 1609 (10.11) | 1614 (13.75) | 2810 (17.25) | 2839 (3.4) | 4282 (6.41) | 5402 (10.08) | 4941 (12.51) | 10164 (17.04) |
| 30-day readmission, N (%**) | 1934 (0.4) | 2071 (0.77) | 1889 (1.35) | 1610 (2.32) | 2835 (4.9) | 1482 (0.33) | 1702 (0.66) | 1650 (1.2) | 1318 (1.93) | 2458 (4.24) | 5584 (0.29) | 5768 (0.57) | 5287 (0.99) | 4572 (1.71) | 8838 (3.84) |

*Among those hospitalized; **Among those discharged alive

Supplementary Table 4: Number and proportion of individuals who experienced each outcome, stratified by continuity of care and number of priority chronic conditions (column percent shown)

|  | High Continuity (COC>0.52) | | | | | Low Continuity (COC≤0.52) | | | | |
| --- | --- | --- | --- | --- | --- | --- | --- | --- | --- | --- |
|  | Number of Priority Chronic Conditions | | | | | Number of Priority Chronic Conditions | | | | |
|  | 1 | 2 | 3 | 4 | 5+ | 1 | 2 | 3 | 4 | 5+ |
| Number of Individuals | 1244438 | 802231 | 430849 | 203662 | 147651 | 1599595 | 745966 | 381580 | 202104 | 200438 |
| Any hospitalization, N (%) | 50082 (4.02) | 47084 (5.87) | 37929 (8.8) | 26063 (12.8) | 30750 (20.83) | 81335 (5.08) | 62276 (8.35) | 49711 (13.03) | 38304 (18.95) | 63052 (31.46) |
| 3+ hospitalizations, N (%*) | 1827 (3.65) | 2506 (5.32) | 2473 (6.52) | 2137 (8.2) | 3276 (10.65) | 3977 (4.89) | 4557 (7.32) | 4858 (9.77) | 4337 (11.32) | 10351 (16.42) |
| Non-medical discharge delay, N (%*) | 1819 (3.63) | 3263 (6.93) | 3740 (9.86) | 3195 (12.26) | 4817 (15.67) | 2760 (3.39) | 4167 (6.69) | 5170 (10.4) | 5120 (13.37) | 11286 (17.9) |
| 30-day readmission, N (%**) | 3024 (0.24) | 3619 (0.45) | 3204 (0.74) | 2579 (1.27) | 3707 (2.52) | 5976 (0.37) | 5922 (0.79) | 5622 (1.48) | 4921 (2.44) | 10424 (5.24) |

*Among those hospitalized; **Among those discharged alive
